# Supranormal Left Ventricular Ejection Fraction, Concentric Remodeling, and Long-Term Survival

**DOI:** 10.1016/j.jacasi.2024.08.020

**Published:** 2024-10-29

**Authors:** Hao-Chih Chang, Chih-Hsueh Tseng, Wei-Ming Huang, Ching-Wei Lee, Wen-Chung Yu, Hao-Min Cheng, Chern-En Chiang, Chen-Huan Chen, Shih-Hsien Sung

**Affiliations:** a Department of Medicine, Taipei Veterans General Hospital Taoyuan Branch, Taoyuan, Taiwan (Republic of China); b Cardiovascular Research Center, College of Medicine, National Yang Ming Chiao Tung University, Taipei, Taiwan (Republic of China); c Institute of Public Health, College of Medicine, National Yang Ming Chiao Tung University, Taipei, Taiwan (Republic of China); d Division of Cardiology, Department of Medicine, Taipei Veterans General Hospital, Taipei, Taiwan (Republic of China); e Institute of Emergency and Critical Care Medicine, College of Medicine, National Yang Ming Chiao Tung University, Taipei, Taiwan (Republic of China); f Division of Holistic and Multidisciplinary Medicine, Department of Medicine, Taipei Veterans General Hospital, Taipei, Taiwan (Republic of China); g Division of Faculty Development, Department of Medical Education, Taipei Veterans General Hospital, Taipei, Taiwan (Republic of China); h General Clinical Research Center, Taipei Veterans General Hospital, Taipei, Taiwan (Republic of China)

**Keywords:** all-cause mortality, concentric remodeling, left ventricular ejection fraction, supranormal left ventricular ejection fraction

## Abstract

**Background:**

Supranormal left ventricular ejection fraction (LVEF) confers a paradoxically higher mortality risk; however, whether intrinsic structural changes of left ventricle (LV) play an important role remain unclear.

**Objectives:**

The authors sought to investigate the prognostic implication of supranormal LVEF and its interaction with LV concentric remodeling.

**Methods:**

Consecutive participants undergoing echocardiography in a tertiary medical center with LVEF >60% were included. LV concentric remodeling was defined as LV relative wall thickness >0.42. The primary outcome was all-cause mortality. The association between LVEF and all-cause mortality was assessed using Cox models and restricted cubic splines. Subgroup analysis was performed to evaluate the association between LVEF and risk of death stratified by LV concentric remodeling.

**Results:**

In total, 67,108 participants (age 60.5 ± 17.2 years, men 44.6% [n = 29,924]) were included. 7,029 deaths of 67,108 (10.5%) occurred over a median of 50.3 months (Q1, Q3: 20.9, 91.3 months). In multivariable Cox models, subjects with LVEF above 70% had a significantly higher risk (vs 60%-65%) for all-cause mortality (adjusted HR: 1.15; 95% CI: 1.05-1.26; *P =* 0.003) after adjusting for potential confounders. A significant interaction was observed between LVEF and LV concentric remodeling (*P* for interaction <0.001), particularly in women, such that a higher mortality risk of supranormal LVEF could be observed mainly among those with LV concentric remodeling.

**Conclusions:**

Supranormal LVEF >70% is associated with a greater risk for all-cause mortality. The higher mortality risk could be predominantly observed among individuals presented with LV concentric remodeling, particularly in women.

Left ventricular ejection fraction (LVEF) is currently the primary parameter to classify patients with heart failure (HF), as well as guidance of clinical therapies.^1^^,^^2^ LVEF also has a prognostic value in predicting clinical outcomes. Evidence has revealed a U-shaped relationship between the spectrum of LVEF and all-cause mortality.^3^^,^^4^ The paradoxically higher mortality risk as LVEF increases has heralded a new phenotype characterized by supranormal LVEF.^3^^,^^5^ However, how the higher risk is attributed to LVEF above the normal range remains unclear. Because that supranormal LVEF contributes to a comparable risk as in those with reduced LVEF, a more clearly defined phenotype of supranormal LVEF is warranted for further investigation on this distinctive entity.

Supranormal LVEF, or hyperdynamic left ventricular (LV) contractility, was initially reported as a surrogate marker for higher mortality risk in critically ill patients.^6^^,^^7^ The higher risk of cardiovascular deaths among subjects with supranormal LVEF was also confirmed later in nationwide register-based studies.^4^ Moreover, in a recent population-based study, supranormal LVEF was found to be associated with excessive cardiovascular events among community-dwelling adults, particularly in those with lower stroke volume.^8^ Whether intrinsic structural changes of LV play an important role in the higher risk of supranormal LVEF remains not well understood. Therefore, in the present study, we aim to examine the association between supranormal LVEF and all-cause mortality, as well as its interaction with LV concentric remodeling in a clinically referred hospital-based cohort.

## Methods

### Study design and study population

We conducted a secondary analysis among ambulatory outpatients who underwent comprehensive echocardiography in a tertiary medical center in Taiwan between April 2005 and December 2021. Consecutive outpatients received their first echocardiography under varying indications, including unknown dyspnea, presumed HF, regular follow-up for certain morbidities, preoperative evaluations, or healthy check-ups. Individuals age ≥20 years and with an LVEF ≥60% were eligible for this study. Subjects with missing LVEF data or with significant valvular heart disease were excluded from analysis. Data on demographic characteristics, body mass index, and comorbidities were prospectively input into a web-based medical recording system. The investigation conformed to the principles outlined in the Declaration of Helsinki. The Institutional Review Committee of Taipei Veterans General Hospital approved the use of the registry data for research purposes and waived the requirement for informed consent.

### Echocardiography assessment

Transthoracic echocardiographic study was conducted according to the recommendations of the American Society of Echocardiography.^9^ All of the echocardiographic images were read by board-certified echocardiographers. LVEF was calculated from the LV end-diastolic volume (EDV) and end-systolic volume (ESV) estimates by biplane Simpson’s method. All LVEFs were categorized into 5% intervals in width. Left atrial (LA) dimension, interventricular septal thickness, and posterior wall thickness were measured using M-mode tracing. Relative wall thickness (RWT) was calculated as: 2 × posterior wall thickness divided by left ventricular internal diameter at end-diastole (LVIDd). LV concentric remodeling was defined as an RWT >0.42. LV mass was calculated as: 0.8 × 1.04 × ([interventricular septal + LVIDd + posterior wall thickness]^3^ − [LVIDd]^3^) + 0.6 grams. Left ventricular mass index (LVMi) was calculated as LV mass divided by body surface area, and left ventricular hypertrophy (LVH) was then defined by an LVMi >115 g/m^2^ in men or >95 g/m^2^ in women. LV end-systolic volume index (ESVi) and end-diastolic volume index (EDVi) were calculated as ESV and EDV divided by body surface area, respectively. E/A ratio represented the ratio of LV early (E) to late (A) filling flow velocity at diastole. E/e’ was the ratio of early ventricular filling flow velocity (E) to septal mitral annulus tissue velocity (e’) at early diastole. Pulmonary artery systolic pressure (PASP) was estimated using Doppler echocardiography by calculating trans-tricuspid pressure gradient during systole and right atrial pressure by the dimension and collapsibility of inferior vena cava.

### Study outcome

The primary outcome was defined as all-cause mortality by linking to the National Death Registry. The National Death Registry is an official and obligatory death registration in Taiwan that registers the date of death and causes of death according to the International Classification of Diseases for all citizens. All study participants were followed up until the death event or the administrative censoring date of December 31, 2022.

### Statistical analysis

Baseline characteristics were described as mean ± SD for continuous variables and percentages for categorical variables. The Student’s *t*-test was used to compare continuous variables, whereas the chi-square test was used to compare categorical variables. Follow-up duration was defined as the length of time between the date of echocardiography and the date of mortality, or the administrative censoring date on December 31, 2022. The Kaplan-Meier method was used to determine cumulative probabilities of death stratified by the categories of LVEF. Multivariable Cox proportional-hazards regression analyses were used to evaluate the association between each group of LVEF (vs LVEF of 60%-65%) and all-cause mortality with adjustment of the theory-driven confounders. HRs, 95% CIs, and *P* values for time-to-event analyses were reported for the primary outcome. The proportional hazards assumption was assessed using the Schoenfeld residual test. No violation was observed for the main independent variable (LVEF groups); however, covariates that violated the proportional hazards assumption were addressed through stratification. Restricted cubic splines were used to demonstrate the association between LVEF as a continuous variable and all-cause mortality. We compared restricted cubic splines models with 3, 4, and 5 knots using the Akaike information criterion and fitted the model with 4 knots at the 5th, 35th, 65th, and 95th percentiles.^10^ We examined the interaction between LVEF and the presence of LV concentric remodeling. Subgroup analyses were performed to assess the consistency of the association between LVEF categories (as an ordinal variable) and all-cause mortality, as well as the interaction between LVEF and LV concentric remodeling among different subgroups (by age, sex, or hypertension) in multivariable models. Sensitivity analyses were conducted to assess the robustness of the findings as follows: 1) exclusion of participants with either septal or posterior wall end-diastolic thickness >13 mm; 2) exclusion of those experiencing early deaths from serious acute illness with follow-up duration <90 days; and 3) stratifying the participants by concentric hypertrophy, defined as RWT >0.42 and the presence of LVH. All statistical analyses were performed using SPSS version 24.0 (SPSS Inc) and R software version 4.0.2 (R Foundation for Statistical Computing). All tests were 2-sided, and *P <* 0.05 was considered statistically significant.

## Results

### Study population

This study included a total of 67,108 participants (age 60.5 ± 17.2 years, 44.6% men [n = 29,924]) who had an LVEF ≥60%. The flowchart of the study population is shown in Supplemental Figure 1. The distribution of LVEF above 60% in our cohort is shown in Supplemental Figure 2. In summary, 41,995 of 67,108 (62.6%) had an LVEF of 60% to 65%, 19,549 of 67,108 (29.1%) had an LVEF of 65% to 70%, 4,012 of 67,108 (6%) had an LVEF of 70% to 75%, and 1,552 of 67,108 (2.3%) had an LVEF >75%. Baseline characteristics between the categories of LVEF were shown in Table 1. Compared with the participants with LVEF of 60% to 65%, individuals with higher LVEF were older and were more likely to have comorbidities, such as hypertension, diabetes, hyperlipidemia, atrial fibrillation, coronary artery disease (CAD), and HF. Among the echocardiographic characteristics, the thickness of interventricular septum and LV posterior wall, LVMi, RWT, average E/e’ ratio, LA dimension, and PASP increased, whereas LV EDVi and ESVi decreased along with the higher LVEF categories.

**Table 1:** Baseline Characteristics Between Individuals by LVEF Subgroups

	LVEF 60%-65% (n = 41,995)	LVEF 65%-70% (n = 19,549)	LVEF 70%-75% (n = 4,012)	LVEF >75% (n = 1,552)	*P* Value^a^
Age, y	59.7 ± 17.3	61.1 ± 16.8^b^	63.1 ± 16.5^b^^,^^c^	65.9 ± 17.1^b^^,^^c^^,^^d^	<0.001
Men	18,964 (45.2)	8,473 (43.3)	1,799 (44.8)	688 (44.3)	0.001
BMI, kg/m^2^	24.4 ± 4.3	24.4 ± 4.2	24.5 ± 4.2	24.6 ± 4.4	0.326
Hypertension	8,867 (21.1)	4,973 (25.4)	1,498 (37.3)	698 (45.0)	<0.001
Diabetes	3,415 (8.1)	1,890 (9.7)	577 (14.4)	279 (18.0)	<0.001
Hyperlipidemia	4,019 (9.6)	2,166 (11.1)	615 (15.3)	255 (16.4)	<0.001
AF	1,359 (3.2)	684 (3.5)	205 (5.1)	101 (6.5)	<0.001
CAD	5,289 (12.6)	2,858 (14.6)	818 (20.4)	363 (23.4)	<0.001
HF	984 (2.3)	547 (2.8)	155 (3.9)	86 (5.5)	<0.001
LVEF, %	62.3 ± 1.3	66.8 ± 1.3^b^	71.8 ± 1.4^b^^,^^c^	77.5 ± 2.5^b^^,^^c^^,^^d^	<0.001
E/A, ratio	1.1 ± 0.5	1.0 ± 0.4^b^	1.0 ± 0.4^b^^,^^c^	1.0 ± 0.5	<0.001
Average E/e’	11.2 ± 4.4	11.4 ± 4.6^b^	11.8 ± 7.4^b^^,^^c^	12.3 ± 5.2^b^^,^^c^^,^^d^	<0.001
LA diameter, mm	35.6 ± 6.6	36.2 ± 6.7^b^	37.3 ± 6.9^b^^,^^c^	37.9 ± 7.6^b^^,^^c^^,^^d^	<0.001
IVST, mm	9.6 ± 2.1	9.8 ± 2.2^b^	10.1 ± 2.5^b^^,^^c^	10.2 ± 2.5^b^^,^^c^	<0.001
PWT, mm	9.5 ± 1.7	9.5 ± 1.8^b^	9.8 ± 2.0^b^^,^^c^	9.8 ± 2.0^b^^,^^c^	<0.001
LVIDd, mm	45.8 ± 5.8	45.6 ± 6.0^b^	46.0 ± 6.3^c^	46.1 ± 6.8^c^	<0.001
EDVi, mL/m^2^	37.9 ± 12.9	37.2 ± 12.6^b^	36.0 ± 11.9^b^^,^^c^	35.2 ± 12.5^b^^,^^c^	<0.001
ESVi, mL/m^2^	14.3 ± 4.9	12.3 ± 4.3^b^	10.1 ± 3.4^b^^,^^c^	7.9 ± 2.9^b^^,^^c^^,^^d^	<0.001
RWT, ratio	0.42 ± 0.09	0.43 ± 0.10^b^	0.44 ± 0.12^b^^,^^c^	0.44 ± 0.13^b^^,^^c^	<0.001
LVMi, gm/m^2^	119.0 ± 56.9	120.1 ± 53.6	125.5 ± 44.3^b^^,^^c^	127.2 ± 44.5^b^^,^^c^	<0.001
PASP, mm Hg	29.2 ± 9.5	29.8 ± 10.0^b^	31.8 ± 11.8^b^^,^^c^	33.5 ± 14.0^b^^,^^c^^,^^d^	<0.001

Values are mean ± SD or n (%).

AF = atrial fibrillation; BMI = body mass index; CAD = coronary artery disease; E/A = the ratio of early to late diastolic transmitral flow velocity; E/e' = the ratio of early diastolic transmitral inflow velocity to early diastolic mitral annular velocity; EDVi = end-diastolic volume index; ESVi = end-systolic volume index; HF = heart failure; IVST = interventricular septal thickness; LA = left atrial; LVEF = left ventricular ejection fraction; LVIDd = left ventricular internal diameter at end diastole; LVMi = left ventricular mass index; PASP = pulmonary artery systolic pressure; PWT = posterior wall thickness; RWT = relative wall thickness.

a Comparison among the 4 groups using 1-way analysis of variance.

b *P <* 0.05 vs LVEF 60%-65%.

c *P <* 0.05 vs LVEF 65%-70%.

d *P <* 0.05 vs LVEF 70%-75%.

Supplemental Table 1 shows the association between comorbidities and LV concentric remodeling. Patients who were older, were men, and had comorbidities, including hypertension, diabetes, hyperlipidemia, and CAD, were more likely to present with LV concentric remodeling. Our data showed that LV concentric remodeling was significantly associated with supranormal LVEF (OR: 1.17 [95% CI: 1.11-1.23]). However, the association was predominantly found in patients with younger age (*P* for interaction < 0.001), without hypertension (*P* for interaction = 0.005), but showed no difference between men and women (*P* for interaction = 0.16) (Supplemental Figure 3).

### Association between supranormal LVEF and risk of mortality

**Figure 1:**
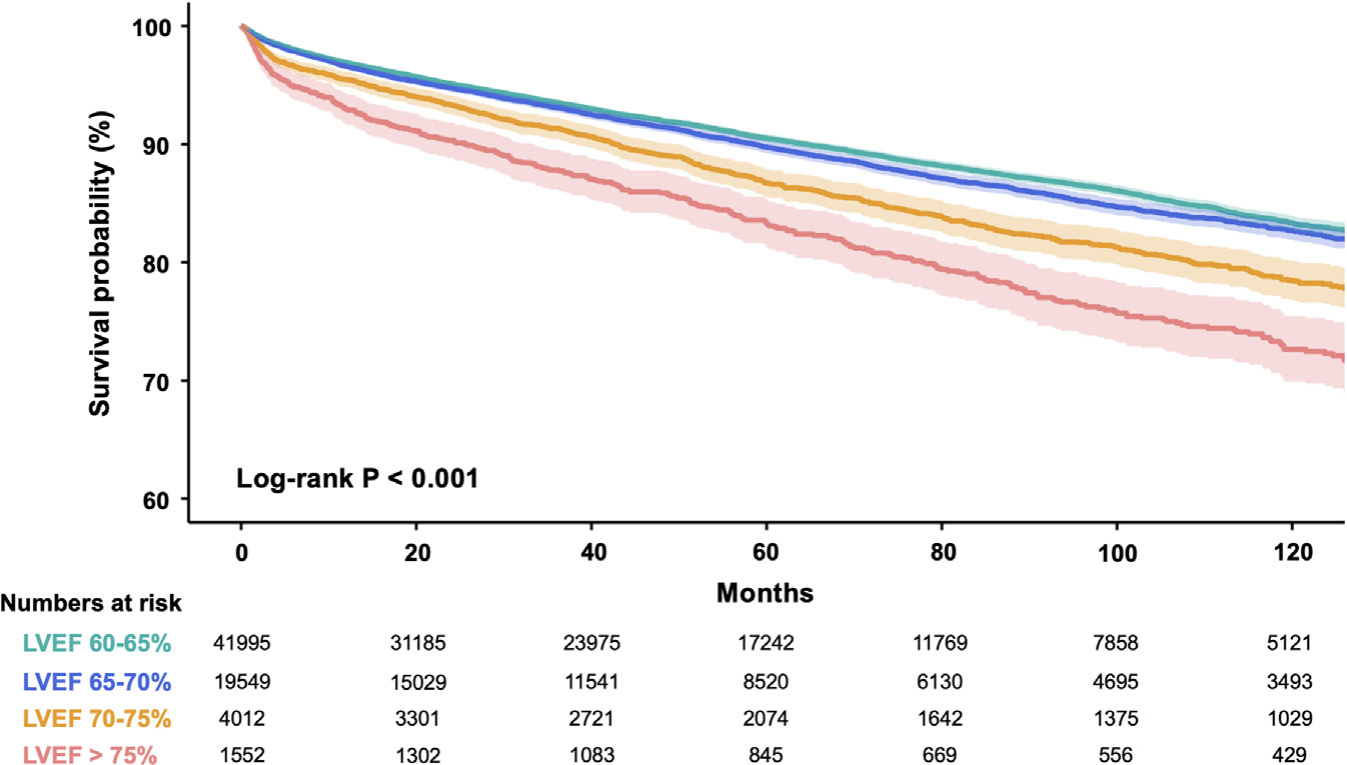
Kaplan-Meier Survival Curves of All-Cause Mortality by LVEF Participants within the subgroup of left ventricular ejection fraction (LVEF) >75% had the highest cumulative probability of all-cause death compared with the other subgroups (log-rank *P <* 0.001).

During a median follow-up of 50.3 months (Q1, Q3: 20.9, 91.3 months), 7,029 of 67,108 participants (10.5%) died. Figure 1 demonstrated the survival probability stratified by different categories of LVEF, and the participants with LVEF >75% were associated with the highest mortality rate (log-rank *P <* 0.001). In the univariate Cox regression analysis, subjects in the higher LVEF categories (65%-70%, 70%-75%, ≥75%) all had a significantly higher mortality than those with LVEF 60% to 65% (Table 2). After accounting for age and sex, participants with LVEF 70% to 75% and ≥75% still had a higher mortality rate than those with LVEF 60% to 65% (HR: 1.25 [95% CI: 1.15-1.36] and 1.43 [95% CI: 1.28-1.60], respectively). In the fully adjusted model, participants with LVEF 70% to 75% and ≥75% remained associated with increased risks of mortality (Table 2). In the pooled cohort analysis of cubic splines evaluating the association between LVEF as a continuous variable and all-cause mortality, a significantly higher risk of all-cause mortality could be observed among patients with higher LVEF (Supplemental Figure 4).

**Table 2:** Association Between LVEF and All-Cause Mortality in Cox Regression Models

LVEF Categories	Crude		Model 1^a^		Model 2^b^	
	HR (95% CI)	*P* Value	HR (95% CI)	*P* Value	HR (95% CI)	*P* Value
LVEF 60%-65%	1.00	Ref.	1.00	Ref.	1.00	Ref.
LVEF 65%-70%	1.07 (1.02-1.13)	0.012	1.04 (0.99-1.10)	0.13	1.00 (0.95-1.06)	0.94
LVEF 70%-75%	1.38 (1.27-1.50)	<0.001	1.25 (1.15-1.36)	<0.001	1.15 (1.05-1.26)	0.003
LVEF >75%	1.81 (1.62-2.02)	<0.001	1.43 (1.28-1.60)	<0.001	1.38 (1.22-1.56)	<0.001

LVEF = left ventricular ejection fraction.

a Model 1: adjusting for age and sex.

b Model 2: adjusting for age, sex, body mass index, diabetes, atrial fibrillation, and heart failure and stratified by hypertension and coronary artery disease.

**Figure 2:**
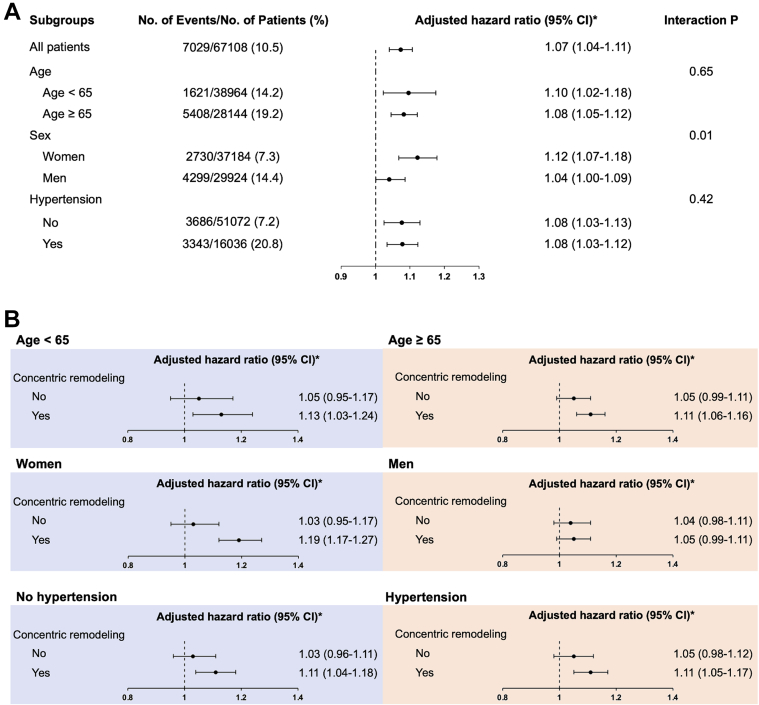
Association Between Supranormal Left Ventricular Ejection Fraction, Mortality, and Interaction With Concentric Remodeling (A) Supranormal left ventricular ejection fraction was consistently associated with a higher mortality risk among all the subgroups; (B) the higher mortality risk could be mainly observed among those with left ventricular concentric remodeling except in men. ∗HR of all-cause mortality for every 5% increase in left ventricular ejection fraction, after adjusting for age, sex, body mass index, hypertension, diabetes, atrial fibrillation, and heart failure.

In the subgroup analysis, supranormal LVEF was consistently associated with a higher risk of all-cause mortality among different groups. However, it was noteworthy that the higher mortality of supranormal LVEF was predominantly shown in women rather than in men (*P* for interaction = 0.02) (Figure 2A).

### Interaction between LVEF and LV concentric remodeling

**Figure 3:**
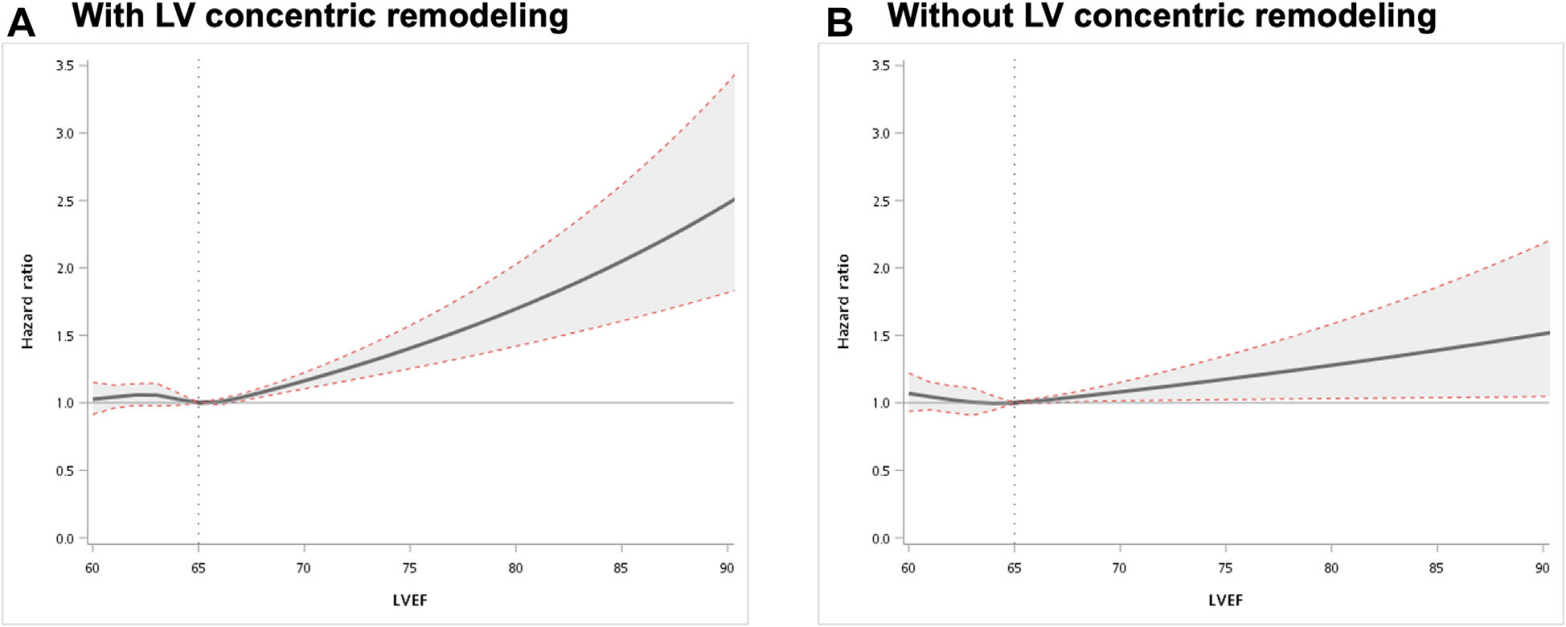
Restricted Cubic Splines on the Association Between LV Ejection Fraction and Mortality (A and B) The association between the continuous measures of left ventricular (LV) ejection fraction and all-cause mortality in the subgroup of individuals with and without concentric remodeling, respectively. Models were adjusted for age, sex, body mass index, hypertension, diabetes, atrial fibrillation, coronary artery disease, and heart failure.

Among the study population, 29,483 of 67,108 participants (43.9%) had LV concentric remodeling. Among subjects with LV concentric remodeling, LVEF 70% to 75% and >75% were associated with increased risks of mortality after adjustment for potential confounders (Table 3). In patients without LV concentric remodeling, only those with LVEF >75% had excessive mortality risks compared with the others. The correlations between LVEF and mortality were predominantly observed in subjects with LV concentric remodeling because the interaction between LVEF and LV concentric remodeling was statistically significant (*P* for interaction < 0.001). Restricted cubic splines also revealed that the association between LVEF and all-cause mortality was steeper among individuals with LV concentric remodeling (Figure 3A) compared with those without LV concentric remodeling (Figure 3B).

**Table 3:** Association Between LVEF and Risk of All-Cause Mortality Stratified By Concentric Remodeling

LVEF Categories	Without Concentric Remodeling^a^			With Concentric Remodeling^a^		
	Events	HR (95% CI)^b^	*P* Value	Events	HR (95% CI)^b^	*P* Value
LVEF 60%-65%	1,617 (6.8)	1.00	Ref.	2,254 (12.5)	1.00	Ref.
LVEF 65%-70%	854 (8.0)	1.01 (0.93-1.11)	0.77	1,226 (14.0)	0.99 (0.92-1.07)	0.78
LVEF 70%-75%	231 (11.3)	1.00 (0.86-1.17)	0.97	407 (21.2)	1.26 (1.12-1.42)	<0.001
LVEF >75%	151 (18.1)	1.27 (1.05-1.53)	0.01	188 (26.7)	1.53 (1.30-1.80)	<0.001

Values are n (%) unless otherwise indicated.

LVEF = left ventricular ejection fraction.

a Concentric remodeling is defined as relative wall thickness >0.42.

b Adjusting for age, sex, body mass index, diabetes, atrial fibrillation, heart failure, and stratified by hypertension and coronary artery disease.

In the subgroup analysis, we further examined the interaction between baseline LVEF and LV concentric remodeling among different subgroups. The positive association between LVEF and mortality risk was consistently observed among subjects with LV concentric remodeling, regardless of age or hypertension, whereas the interaction between LVEF and LV concentric remodeling was primarily observed in women but not in men (Figure 2B).

### Sensitivity analysis

When we excluded participants with either interventricular septum or posterior wall of >13 mm (n = 4,106) to expel the potential diagnosis of hypertrophic cardiomyopathy, a significantly higher risk for all-cause mortality among participants with supranormal LVEF and concentric remodeling could be still observed (Supplemental Figure 5A). To discount severely ill subjects, we excluded participants with follow-up duration <90 days (n = 5,078). Again, the correlation between supranormal LVEF and mortality remained, regarding LV concentric remodeling (Supplemental Figure 5B). With further stratification of LVH, the positive association between LVEF and mortality risk could be primarily observed in those with LV concentric hypertrophy (Supplemental Figure 5C).

## Discussion

In this large, hospital-based cohort study with a long-term follow-up, we found that participants with supranormal LVEF >70% had a significantly higher risk for all-cause mortality, independent of traditional cardiovascular risk factors. The association between supranormal LVEF and the risk of mortality could be modified by the presence of LV concentric remodeling. That is, the higher long-term mortality risk was predominantly observed among participants with LV concentric remodeling, rather than in those without LV concentric remodeling. The interaction between LVEF and LV concentric remodeling was observed more in women (Central Illustration). Our study provided further clinical evidence for a more clearly defined phenotype of supranormal LVEF that may facilitate further investigation on this distinctive high-risk entity.

**Central Illustration:**
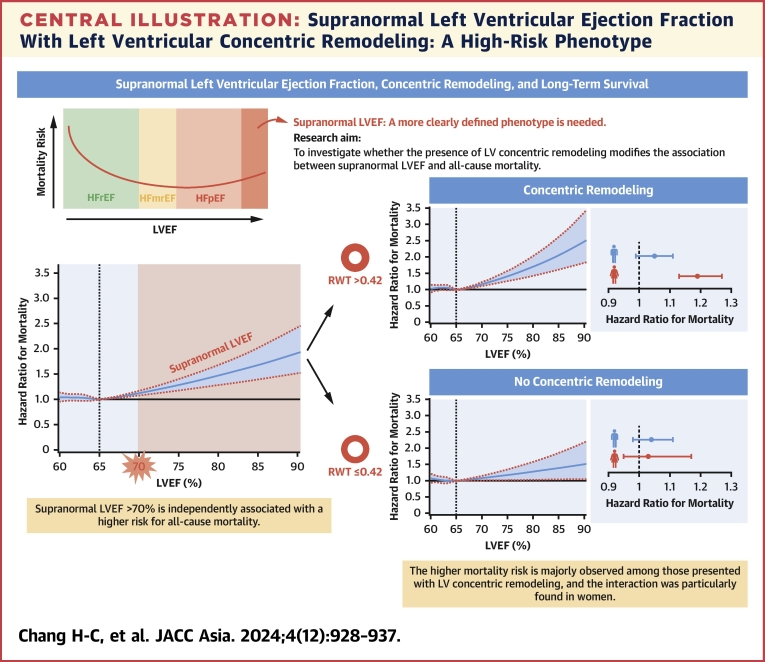
Supranormal Left Ventricular Ejection Fraction With Left Ventricular Concentric Remodeling: A High-Risk Phenotype Supranormal LVEF of more than 70% is independently associated with a greater mortality risk, and the higher mortality risk can be predominantly observed among women who presented with LV concentric remodeling. HFmrEF = heart failure with mildly reduced ejection fraction; HFpEF = heart failure with preserved ejection fraction; HFrEF = heart failure with reduced ejection fraction; LV = left ventricular; LVEF = left ventricular ejection fraction; RWT = relative wall thickness.

Because a U-shaped relationship between LVEF and risk of all-cause mortality has been consistently observed in previous studies, the paradoxically higher mortality risk among those with increased LVEF suggested a novel phenotype of supranormal LVEF.^3^^,^^4^ Individuals with supranormal LVEF could even carry a comparable risk as those with an LVEF of 35% to 40%.^3^ Therefore, a clearly defined absolute cutoff value for supranormal LVEF is necessary to separate this distinctive phenotype from the heterogeneous pool of individuals with preserved LVEF and to facilitate further investigation on how to treat this paradoxically vulnerable subgroup. Our study revealed that supranormal LVEF >70% expressed a significantly increased mortality risk compared with those with normal LVEF. The significant interaction between LVEF and LV concentric remodeling found in our study indicated that both LVEF and morphological changes of LV should be assessed to identify the higher-risk subgroup among supranormal LVEF.

The underlying mechanisms of the paradoxically increased mortality risk in individuals with supranormal LVEF have yet to be elucidated. Prior study identified 16 genetic variants implicating for cardiac hypertrophy from a genome-wide association study that were associated with supranormal LVEF and increased risk of mortality.^11^ In a population-based cohort study enrolling community-dwelling adults, Shah et al^8^ observed that higher LVEF was associated with a greater major adverse cardiovascular event only among those with reduced LV stroke volume index. Compatible with previous study, our study could also find that the higher mortality risk of supranormal LVEF was predominantly found among those with reduced LV stroke volume index (*P* for interaction <0.001) (Supplemental Table 2). Our study provided further evidence that the presence of LV concentric geometry might help identify individuals with supranormal LVEF that correlated with higher downstream risk. Compared with the LV stroke volume index, LV concentric remodeling could be more readily assessed by measuring RWT using echocardiography in clinical practice. The measurement of RWT is less susceptible to potential pitfalls, such as varying loading conditions or arrhythmias,^12^ and benefits from the established threshold of 0.42 for defining LV concentric remodeling.^9^ The present study supported that intrinsic morphological changes of LV could be a risk marker of the higher risk of supranormal LVEF. On the contrary, participants with commensurate supranormal LVEF but without concentric remodeling, characterized as normal LV wall thickness, less decreased LV chamber size, lower average E/e’ ratio, normal LA dimension, and lower PASP, can represent the comparative phenotype with lower risk profile (Supplemental Table 3).

It is also noteworthy that sex-based differences could be observed in the association between supranormal LVEF and all-cause mortality, as well as the interaction between LVEF and LV remodeling from our study. In accordance with prior publications enrolling patients with CAD or hospitalized for HF, women with increased LVEF (>65%) were also found to have a higher risk for cardiovascular events or death than men.^13^, ^14^, ^15^ The smaller LV volumes with compromised pumping efficiency, the more microvascular dysfunction and an increased sympathetic tone in women were the proposed mechanisms that may contribute to the higher risk of supranormal LVEF in women.^13^^,^^14^^,^^16^^,^^17^ On the contrary, the larger ESVi observed in men might provide a compensatory mechanism against the negative impacts of LV concentric remodeling compared with women.^18^ Among patients with supranormal LVEF, our data showed that men with LV concentric remodeling could have an even larger ESVi than women without LV concentric remodeling (9.9 ± 3.4 mL/m^2^ vs 9.6 ± 3.1 mL/m^2^; *P =* 0.20), whereas women with LV concentric remodeling had the smallest ESVi (8.4 ± 2.7 mL/m^2^; *P <* 0.001). The larger ESVi might provide a protective mechanism for men against the detrimental effects of LV concentric remodeling.^16^^,^^17^ However, limited by the cross-sectional evaluation of echocardiography, a further longitudinal follow-up study will be required to investigate the trajectory of the change in LVEF to explore the underlying mechanisms of its poorer prognosis.

Although supranormal LVEF confers a significant risk for cardiovascular events, there is a lack of clinical trials investigating the efficacy of current guideline-directed medical therapies in this specific population. In the subgroup analyses from recent clinical trials of either angiotensin receptor-neprilysin inhibitor or sodium-glucose cotransporter-2 inhibitors in patients with preserved LVEF, the treatment effects could be inconsistent in the subgroups of higher LVEF from different trials.^19^, ^20^, ^21^ Interpretation of these results could be limited by the inconsistent cutoff values categorizing LVEF above 50% among different trials, as well as limited by the nature of post hoc subgroup analysis. To investigate the efficacy of current foundational therapies among individuals with supranormal LVEF, further prospective clinical trials using a more clear or generally accepted definition of supranormal LVEF will be warranted.^22^^,^^23^

### Study strength and limitations

To the best of our knowledge, this study was the first to investigate the interaction between LVEF and LV concentric remodeling on long-term outcome among subjects with supranormal LVEF. The key strength of the present study included a large sample size with comprehensive echocardiographic measurements and with long-term follow-up. However, there are some limitations that need to be addressed. First, considering this single-center cohort study enrolled only Asian individuals, the results may have limited generalizability to other ethnic groups. Second, although we have adjusted all of the available relevant variables in the multivariable model, unmeasured confounders still may have existed. Third, because LV concentric remodeling was primarily determined by RWT in the present study, the measurement of RWT could be overestimated by 2-dimensional echocardiography.^24^ However, sensitivity analysis stratifying participants with or without LV concentric hypertrophy showed a similar pattern that further consolidated our main findings. However, further validation using 3-dimensional echocardiography or cardiac magnetic resonance imaging may still be needed.

## Conclusions

Supranormal LVEF of >70% was independently associated with an increased risk for long-term mortality, particularly in those that presented with LV concentric remodeling. The interaction between LVEF and LV concentric remodeling was predominantly observed in women. Supranormal LVEF should be considered as a clinically distinctive phenotype from the heterogeneous pool of individuals with preserved LVEF. Further research is essential to clarify a well-defined phenotype of supranormal LVEF, which will be valuable for future clinical trials focusing on preventive and therapeutic strategies for this distinctive entity.

Perspectives **CLINICAL COMPETENCIES:** Supranormal LVEF should be considered a clinically distinctive phenotype from the heterogenous pool of preserved LVEF. Supranormal LVEF of more than 70% was independently associated with a greater risk of death, particularly among women presented with LV concentric remodeling. LV intrinsic structural change should be considered a risk marker of the higher mortality risk among individuals with supranormal LVEF.

**TRANSLATIONAL OUTLOOK:** A clearly defined phenotype of supranormal LVEF can help guide further clinical trials on preventive and therapeutic strategies for this distinctive entity.

## Funding Support and Author Disclosures

This work received support from Ministry of Health and Welfare, Taiwan (grant numbers MOHW110-TDU-B-211-124001, MOHW111-TDU-B-211-134001, MOHW112-TDU-B-211-144001), National Science and Technology Council Science and Technology of Taiwan (grant numbers NSTC111-2314-B-A49 -010, NSTC112-2314-B-A49 -042), Taipei Veterans General Hospital-National Taiwan University Hospital Joint Research Program (grant numbers VN111-04, VN112-05), and Taipei Veterans General Hospital (grant numbers V107C-027, V108-133). The authors have reported that they have no relationships relevant to the contents of this paper to disclose.
