# Supplementary material for: Supranormal Left Ventricular Ejection Fraction, Concentric Remodeling, and Long-Term Survival
Source: JACC Asia. 2024 Oct 29;4(12):928–37. doi: 10.1016/j.jacasi.2024.08.020 (PMC11711995; doi:10.1016/j.jacasi.2024.08.020)
Supplement: Supplemental Figures 1-5 and Supplemental Tables 1-3 [file mmc1.docx]

**Supranormal Left Ventricular Ejection Fraction, Concentric Remodeling, and Long-Term Survival**

**Running title:** Supranormal LVEF and LV concentric remodeling

Hao-Chih Chang^1,2,3^, MD; Chih-Hsueh Tseng^4,5,6^, MD; Wei-Ming Huang^3,4^, MD, PhD; Ching-Wei Lee^4^, MD; Wen-Chung Yu^2,4^, MD; Hao-Min Cheng^2,3,7^, MD, PhD; Chern-En Chiang^2,4,8^, MD, PhD; Chen-Huan Chen^2,3^, MD; Shih-Hsien Sung^2,3,4^*, MD, PhD.

**SUPPLEMENTAL MATERIAL**

**Supranormal left ventricular ejection fraction, concentric remodeling, and long-term survival**

Hao-Chih Chang^1,2,3^, MD; Chih-Hsueh Tseng^4,5,6^, MD; Wei-Ming Huang^3,4^, MD, PhD; Ching-Wei Lee^4^, MD; Wen-Chung Yu^2,4^, MD; Hao-Min Cheng^2,3,7^, MD, PhD; Chern-En Chiang^2,4,8^, MD, PhD; Chen-Huan Chen^2,3^, MD; Shih-Hsien Sung^2,3,4^*, MD, PhD.

^1^ Department of Medicine, Taipei Veterans General Hospital Taoyuan Branch, Taoyuan, Taiwan (R.O.C.);

^2^ Cardiovascular Research Center, College of Medicine, National Yang Ming Chiao Tung University, Taipei, Taiwan (R.O.C.);

^3^ Institute of Public Health, College of Medicine, National Yang Ming Chiao Tung University, Taipei, Taiwan (R.O.C.);

^4^ Cardiovascular Center, Taipei Veterans General Hospital, Taipei, Taiwan (R.O.C.);

^5^ Institute of Emergency and Critical Care Medicine, College of Medicine, National Yang Ming Chiao Tung University, Taipei, Taiwan (R.O.C.);

^6^ Division of Holistic and Multidisciplinary Medicine, Department of Medicine, Taipei Veterans General Hospital, Taipei, Taiwan (R.O.C.);

^7^ Division of Faculty Development, Department of Medical Education, Taipei Veterans General Hospital, Taipei, Taiwan (R.O.C.);

^8^ General Clinical Research Center, Taipei Veterans General Hospital, Taipei, Taiwan (R.O.C.).

*** Information for correspondence:**

Shih-Hsien Sung, M.D., Ph.D.

Institute of Emergency and Critical Care Medicine, National Yang Ming Chiao Tung University.

No. 155, Sec. 2, Linong Street, Beitou District, Taipei 112304, Taiwan (R.O.C.).

Tel: +886-2-2821-1699; Fax: +886-2-2820-2190.

E-mail: [mr.sungsh@gmail.com](mailto:mr.sungsh@gmail.com)

**Supplemental Figure 1. Flowchart of the study population.**


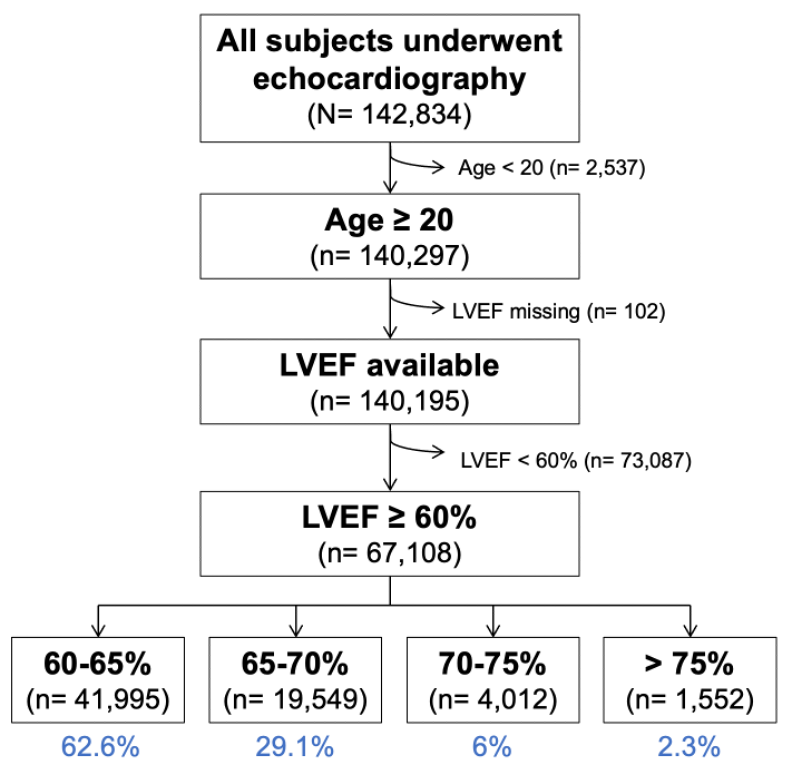


LVEF, left ventricular ejection fraction.

**Supplemental Figure 2. Histogram distribution of LVEF in the study cohort.**

**
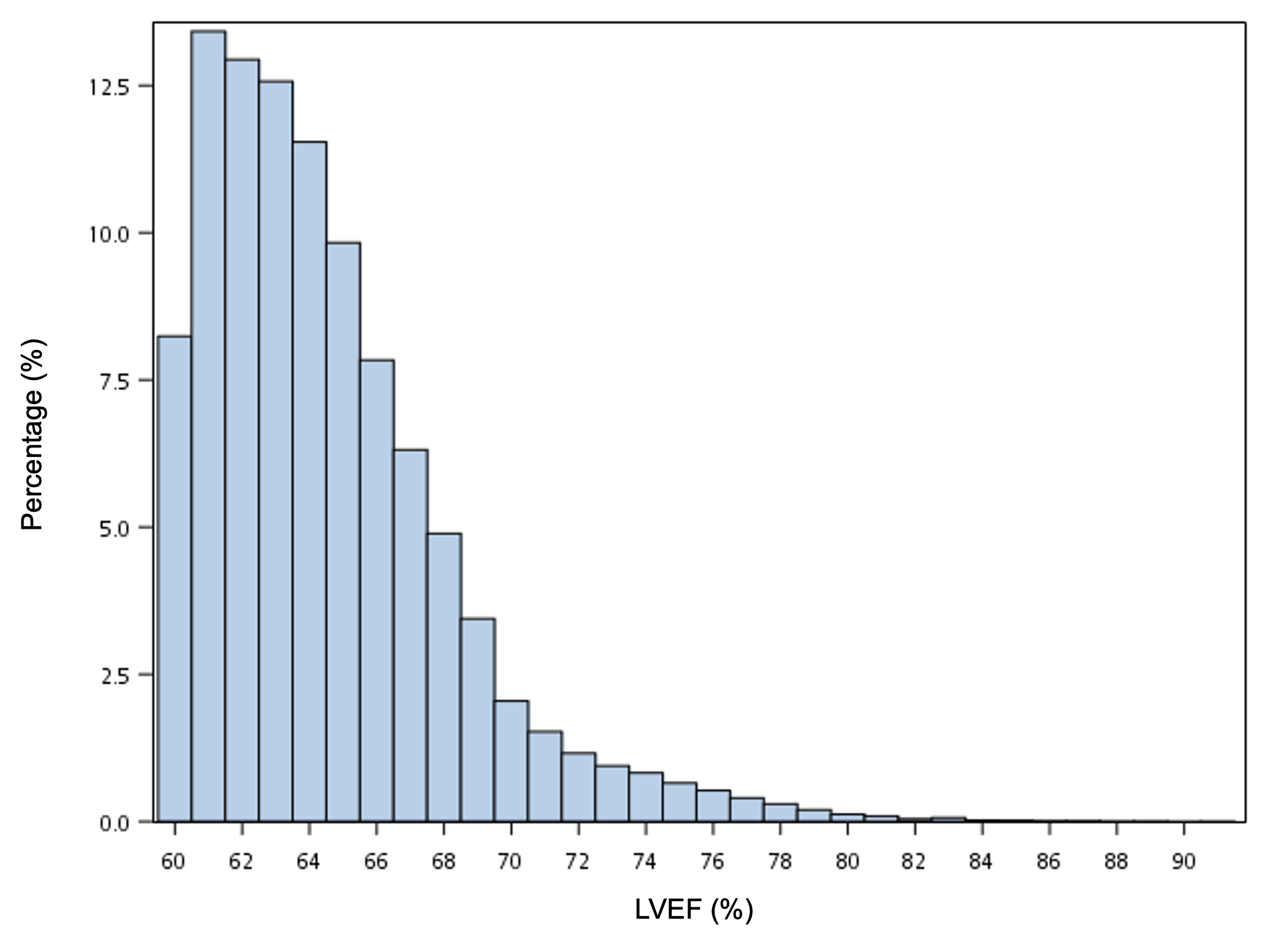
**

LVEF, left ventricular ejection fraction.

**Supplemental Figure 3. Association between LV concentric remodeling and supranormal LVEF.**

**
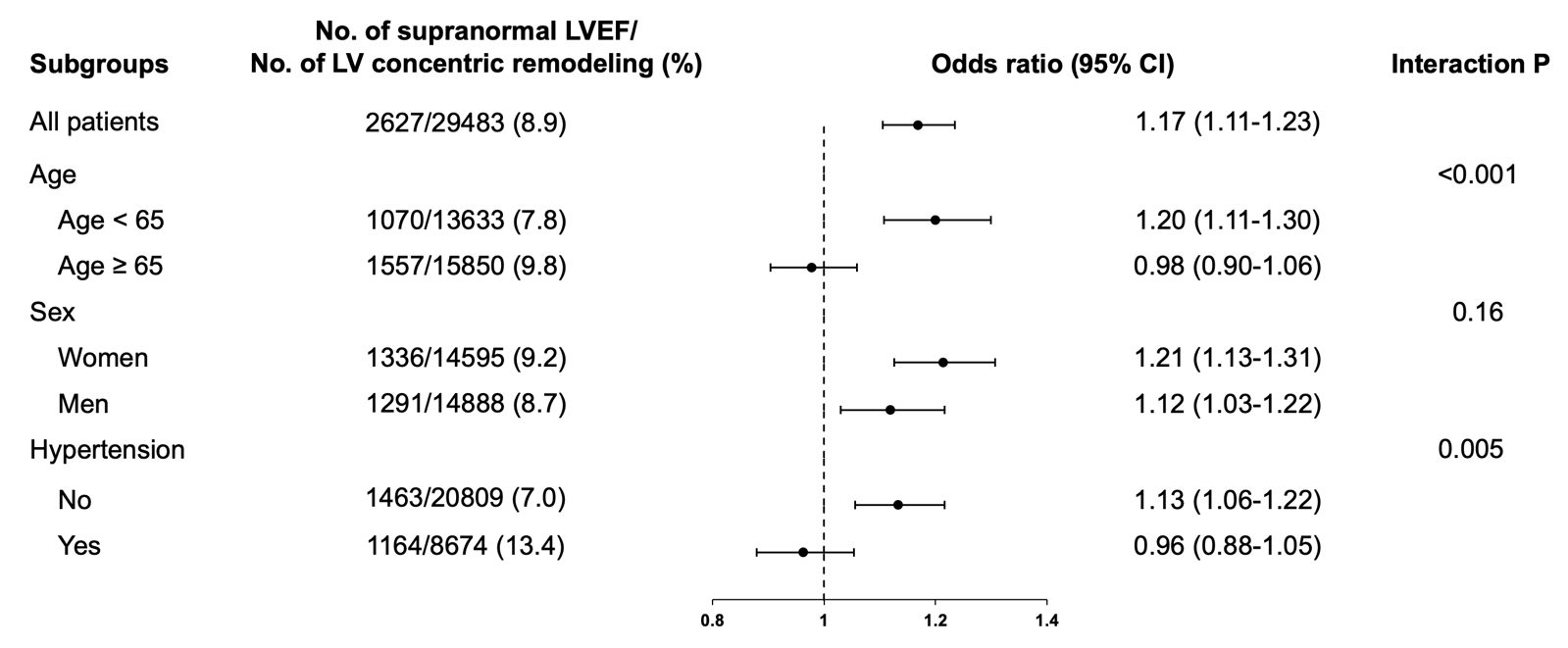
**

Abbreviations: CI, confidence interval; LV, left ventricular; LVEF, left ventricular ejection fraction.

**Supplemental Figure 4. Restricted cubic splines on the association between LVEF and all-cause mortality.**


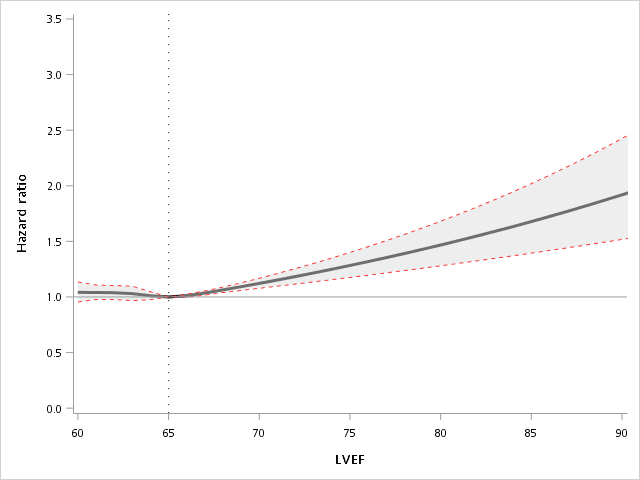


LVEF, left ventricular ejection fraction.

**Supplemental Figure 5. Sensitivity analyses of the interaction between LVEF and concentric remodeling on all-cause mortality.**


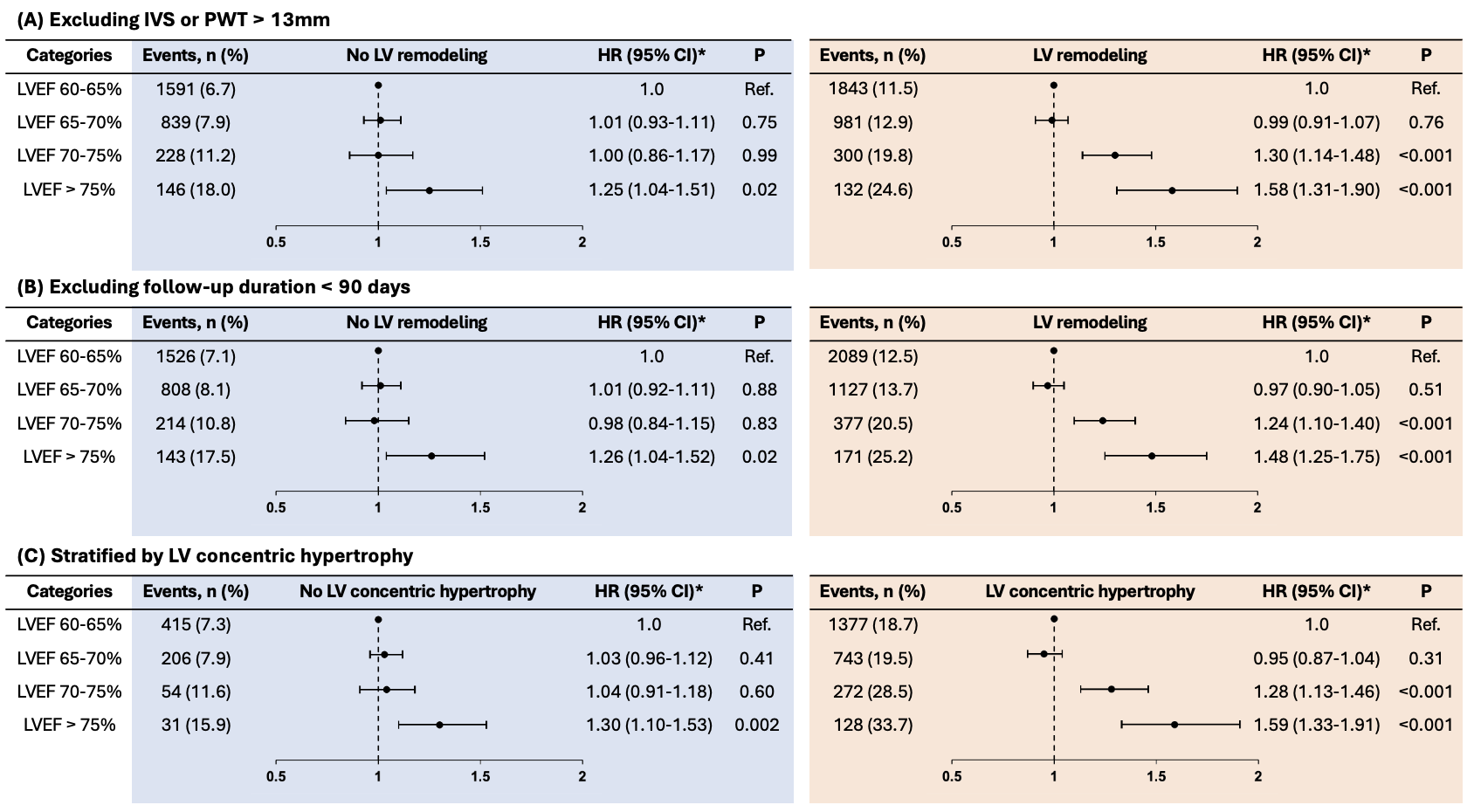


Panel A shows the relationship between LVEF and all-cause mortality, after exclusion of participants with either left ventricular interventricular septum or posterior wall thickness of more than 13 mm. Panel B shows the relationship between LVEF and all-cause mortality, after exclusion of participants with follow-up duration less than 90 days. Panel C shows the relationship between LVEF and all-cause mortality stratified by the presence of concentric hypertrophy or not. Concentric hypertrophy was defined as relative wall thickness > 0.42 and increased LVMi (men > 115 gm/m^2^ or women > 95 gm/m^2^).

* Adjusting for age, sex, body mass index, diabetes, atrial fibrillation, heart failure and stratified by hypertension and coronary artery disease.

LVEF, left ventricular ejection fraction; LVMi, left ventricular mass index.

**Supplemental Table 1. Association between demographics, comorbidities, and LV concentric remodeling.**

| **Variables** | **OR (95% CI)** | **P-value** |
| --- | --- | --- |
| Age | 1.036 (1.035-1.037) | <0.001 |
| Men | 1.543 (1.496-1.591) | <0.001 |
| BMI | 1.045 (1.040-1.050) | <0.001 |
| Hypertension | 1.739 (1.678-1.803) | <0.001 |
| Diabetes | 1.892 (1.794-1.996) | <0.001 |
| Hyperlipidemia | 1.370 (1.304-1.440) | <0.001 |
| CAD | 1.212 (1.160-1.266) | <0.001 |
| Abbreviations: BMI, body mass index; CAD, coronary artery disease; CI, confidence interval; OR, odds ratio. | | |

**Supplemental Table 2. Association between LVEF with risk of all-cause mortality stratified by LV stroke volume index.**

| **LVEF categories** | **LV stroke volume index above median** | | | **LV stroke volume index below median** | | | |
| --- | --- | --- | --- | --- | --- | --- | --- |
|  | **Events, n (%)** | **HR (95% CI)^†^** | **P-value** | **Events, n (%)** | **HR (95% CI)^†^** | **P-value** |  |
| LVEF 60-65% | 1005 (11.3) | 1.0 | Ref. | 1264 (12.6) | 1.0 | Ref. |  |
| LVEF 65-70% | 496 (11.3) | 0.99 (0.89-1.10) | 0.80 | 532 (14.5) | 1.03 (0.93-1.14) | 0.53 |  |
| LVEF 70-75% | 102 (13.3) | 1.03 (0.84-1.26) | 0.80 | 122 (22.3) | 1.48 (1.23-1.78) | <0.001 |  |
| LVEF >75% | 59 (21.3) | 1.46 (1.12-1.90) | 0.005 | 46 (28.2) | 1.78 (1.33-2.40) | <0.001 |  |
| **†** Adjusting for age, sex, body mass index, hypertension, diabetes, atrial fibrillation, coronary artery disease, heart failure.  Abbreviations: CI, confidence interval; HR, hazard ratio; LV, left ventricular; LVEF, left ventricular ejection fraction. | | | | | | |  |

**Supplemental Table 3. Comparison of echocardiographic indices in participants with supranormal LVEF (> 70%): with vs. without concentric remodeling.**

| **Variables** | **No concentric remodeling**  **RWT ≤ 0.42**  **(n= 2,885)** | **Concentric remodeling**  **RWT > 0.42**  **(n= 2,627)** | **P-value** |
| --- | --- | --- | --- |
| LVEF, % | 73.4±3.1 | 73.4±3.1 | 0.73 |
| IVST, mm | 9.0±2.0 | 11.5±2.4 | <0.001 |
| PWT, mm | 8.6±1.2 | 11.1±1.8 | <0.001 |
| LVIDd, mm | 48.8±5.4 | 42.9±6.1 | <0.001 |
| EDVi, ml/m^2^ | 37.8±12.7 | 34.3±11.3 | <0.001 |
| ESVi, ml/m^2^ | 10.1±3.6 | 9.1±3.2 | <0.001 |
| RWT, ratio | 0.4±0.04 | 0.5±0.1 | <0.001 |
| LVMi, gm/m^2^ | 118.3±41.7 | 133.7±45.6 | <0.001 |
| E/A, ratio | 1.1±0.5 | 0.9±0.3 | <0.001 |
| Average E/e’ | 11.1±7.7 | 12.9±5.7 | <0.001 |
| LA diameter, mm | 36.7±6.9 | 38.4±7.2 | <0.001 |
| PASP, mmHg | 31.2±11.6 | 33.5±13.2 | <0.001 |
| Moderate/severe MR, n (%) | 128 (4.4) | 99 (3.8) | 0.21 |
| Abbreviations: E/A, the ratio of early to late diastolic transmitral flow velocity; E/e', the ratio of early diastolic transmitral inflow velocity to early diastolic mitral annular velocity; EDVi, end-diastolic volume index; ESVi, end-systolic volume index; IVST, interventricular septal thickness; LA, left atrial; LVEF, left ventricular ejection fraction; LVIDd, left ventricular internal diameter at end diastole; LVMi, left ventricular mass index; MR, mitral regurgitation; PASP, pulmonary artery systolic pressure; PWT, posterior wall thickness; RWT, relative wall thickness. | | | |
